# Special Characteristics of Alpha Generation Children Behavior in Dentistry: A Literature Review

**DOI:** 10.1055/s-0043-1776336

**Published:** 2024-01-10

**Authors:** Jose Mario Hutajulu, Hendriati Agustiani, Arlette Suzy Setiawan

**Affiliations:** 1 Dental Education Program, Faculty of Dentistry, Universitas Padjadjaran, Bandung, Indonesia; 2 Department of Developmental Psychology, Faculty of Psychology, Universitas Padjadjaran, Bandung, Indonesia; 3 Department of Pediatric Dentistry, Faculty of Dentistry, Universitas Padjadjaran, Bandung, Indonesia

**Keywords:** generation alpha, characteristics, behavior, technology, dentistry, behavior management

## Abstract

Technological developments and advances have influenced the emergence of a new generation, known as Generation Alpha. This generation comprises those born between 2010 and 2025. Born into a digital-savvy era, this generation has different characteristics from previous generations. This study aims to identify their special characteristics so that an approach can be taken, especially in managing children of the alpha generation in dentistry. A systematic search for articles, published between 2013 and 2023 analyzing the characteristics and behavior of the alpha generation and management behavior found in dentistry was conducted through PubMed, Google Scholar, SCOPUS, and EBSCO. The final analysis was carried out on 47 articles consisting of 10 articles discussing the characteristics of the alpha generation in general and 37 articles discussing the management of their behavior in dentistry. All the published articles found that an alpha child's characteristics are closely related to behavior management in dentistry. The ease with which alpha children adapt to technology is one of the strategies for managing the behavior of alpha children. However, apart from this, the alpha children tend to be unappreciative of the process, which can affect dental behavior management. Specific characteristics of the alpha generation, such as increased exposure to technology, digital media consumption habits, and lack of respect for the process, have important implications for communication and adaptation to patient behavior management in dentistry. Understanding these characteristics is crucial for designing an effective communication strategy and adjusting appropriate behavior management to maintain the quality of dental care for this generation in the dentistry environment.

## Introduction

Advances in technology significantly affect the newest generation, which tends to be more interested in technology and the digital world. 1 The Alpha generation (the Glass Generation), born in 2010 and after, is the newest generation and is expected to fill elementary school classrooms with technology. They are projected to be the largest generation to be born in 2025. They will also soon be entering the classroom in higher education, which requires an integrated approach based on their characteristics. A unique approach that can be taken is using technology such as audio, visual, and kinesthetic tools that support learning and daily activities because this alpha generation is very connected and dependent on technology. 2 3 4

The alpha generation is intelligent and is considered more robust in its critical vision. 2 This shows that the alpha generation can deconstruct and analyze ideas, so it is assumed that they will use rational thinking more than feelings. It is supported that the alpha generation prioritizes technology in everyday life, such as using audio/visual tools to communicate and seek information. This can result in a lack of social interaction except through technology. These characteristics indicate that the alpha generation is more focused on the thoughts that shape their character, which makes them psychological, especially in the intellectual and behavioral aspects. Therefore, the results of the traits and behaviors found from the intellectual and behavioral aspects will determine their cooperation in specific fields. Related parties must be able to adapt to be able to interact and connect directly with this generation. 1 2 3

Behavior is the dominant factor in dentistry affecting dental and oral health status. 5 Dentists need to understand the characteristics and behavior because patients come to the dentist based on the dimensions of the oral health-related quality of life that apply to pediatric patients regardless of whether they have oral health problems now or in the future so that they get satisfactory treatment results. 6 Children's dental and oral care behavior is influenced by parental factors, the dental team, dental clinic environment, and the child's factor, which is the most crucial factor. 7 Therefore, it is essential to characterize the behavior of these alpha children as they will represent the future and provide a lens through which to view the next decade and beyond. In the current era, pediatric dentistry is a field that must evolve to face the progress of the era, especially with the birth and existence of this generation of alpha children; it must be prepared for their presence in clinics and need to adapt to be able to use more targeted methods. 3

Based on the results of studies that have been done before, no literature study has discussed the characteristics of the alpha generation and its relation to dentistry. Therefore, it is necessary to study the characteristics of the alpha generation and its relation to dentistry to be used as an initial step and complementary information to facilitate future research. The purpose of this study was to review existing research on the specific characteristics of alpha child behavior and its relation to dental clinics.

## Methods

The type of study used is literature study with the method of traditional literature study or narrative review. In this study, the author systematically searches and selects articles by correlating joint studies on a topic to develop or review articles. The study was conducted from January to May 2023 by accessing research articles on human subjects published in national and international journals through academic databases PubMed, Google Scholar, SCOPUS, and EBSCO. The search result data are documented using the Reference Manager Application (Mendeley).

### Research Question and Selection of Keywords

Determining research questions is our first step in conducting this literature study. According to the Population, Intervention, Comparison, and Outcome (PICO) structure, the formulation of the research question will be as follows: P = alpha generation children; I = nonpharmacological approach; C = does not apply; and O = behavior in dentistry. “In Alpha Generation children (P), what are the effects of nonpharmacological techniques (I) on behavior during dental visits (O)?”

### Searching Strategy

The search was carried out by identifying journals according to the research topic. In this study, the electronic databases used were PubMed, Google Scholar, SCOPUS, and EBSCO by applying keywords, inclusion, and exclusion categories. The keywords used were words or phrases related to the characteristics and behavior of children of the alpha generation and behavior management in dentistry. Boolean operators with the following keywords were used: “Alpha generation” AND “Dentistry” AND “Behavior management”; “non-pharmacological techniques” OR “Behavioral interventions”; “Children” NOT “Adults.”

### Inclusion Criteria and Exclusion Criteria

The inclusion criteria used in the electronic database were (1) articles published within the last 10 years, (2) articles in English or Indonesian, (3) research involving humans (alpha generation children) as research subjects on the management of children in dentistry, (4) articles discussing the characteristics of alpha generation children, and (5) research that included observation and review. The exclusion criteria in this study were (1) articles that did not examine the characteristics and behavior of children of the alpha generation, (2) articles that did not examine the management behavior of children of the alpha generation in dentistry, and (3) articles with subjects who had a history of significant developmental disorders or severe conditions.

### Data Extraction

The data obtained were extracted in several tables, namely, the general characteristics table of 10 articles, which reviewed the general characteristics of alpha generation children, and a summary table of 37 articles, which examined the relationship between unique characteristics of alpha generation children and behavior management in dentistry.

## Result

### Article Selection Results

**Fig. 1:**
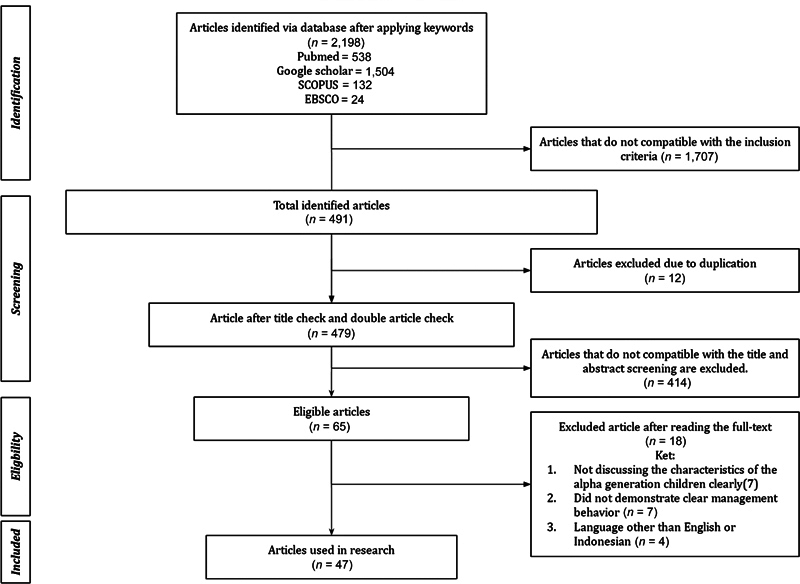
Article selection flowchart.

The results of the study show the main findings of the research. The author obtained 2,198 articles consisting of 538 articles through PubMed, 1,504 articles from Google Scholar, 132 from SCOPUS, and 24 from EBSCO. Then filtering was carried out based on the title so that 1,707 articles were filtered and 491 articles were obtained. After checking several articles, 12 were deleted, resulting in 479 articles for further identification. Based on the examination of titles and abstracts, the authors screened again so that 65 articles were obtained, which would be assessed as a whole. Eighteen articles that did not meet the inclusion criteria were excluded, with details of 7 articles not discussing the characteristics of the alpha generation of children, 7 articles not showing management behavior in dentistry, and 4 articles not in English or Indonesian. In the end, 47 articles were obtained that met the criteria and were eligible to be included in this study. The results of the article selection are summarized in the article selection flowchart ( Fig. 1 ).

### Article Characteristics

The general characteristics of the articles in Table 1 include a description of the research title, country and name of the researcher, year of publication of the article, research design and number of samples, research objectives, research findings, and a brief conclusion from the research articles included. All articles used qualitative research ( *n* = 10), with most of the research ( *n* = 7) using a literature study design, 1 8 9 10 11 12 13 one article ( *n* = 1) using a qualitative descriptive research design, 14 one article ( *n* = 1) using a qualitative research design with content analysis, 15 and one study ( *n* = 1) using a qualitative research design with theoretical analysis. 4 The complete characteristics of the article are listed in Table 1 .

**Table 1:** Presentation of the overview of 10 articles that examine the characteristics of alpha generation children

Sl. no.	Title	Study	Study design and samples	Objective	Findings of the characteristics of alpha generation children	Conclusion
1	Memahami Perkembangan Anak Generasi Alfa di Era Industri 4.0	Fadlurrohim et al 8	Literature study	Understanding the development of alpha generation children in the industrial era 4.0	1. The generation most familiar with digital technology 2. The generation that is claimed to be the smartest 3. The generation most familiar with the internet of all time 4. McCrindle 56 also predicts that the alpha generation cannot be separated from gadgets, lacks socialization and creativity, and is individualistic. 5. Wanting instant things and not appreciating the process. Their preoccupation with gadgets makes them socially alienated 6. Having a skill that is shown (become more specialized) has a specialization 7. Lack of activities, drink less and rarely leave the house but spend a lot of time online	Generation Alpha has a close relationship with digital technology. It has intelligent characteristics but has several challenges, such as dependence on gadgets and deficiencies in social interaction and activities outside the home
2	Visual literacy and character education for alpha generation	Ramadlani and Wibisono 14	Qualitative descriptive	Knowing the application of alpha generation visual literacy strategies	According to research conducted by Barkowitz (2016) 13 64 , namely: 1. Antisharing in the economy because of the behavior shown by this generation 2. Generation is very active, except in a stationary position 3. Lack of privacy concern, tend to overexpose themselves 4. Less fond of rules, tend to violate existing rules 5. Seeking freedom from limitations tends to avoid all kinds of restrictions 6. Prefer natural and fresh milk for health 7. Likes biscuits, pasta, rice, cereals, and vitamins 8. Feeling uncomfortable in formal religious rituals tends to disrupt events 9. Innovative in using old parents' items 10. Prefers a soft touch over touchscreens and tends to lick things to feel the sensation 11. Prefer something familiar, don't like complicated dramas or serials. 12. Living in the moment, paying little attention to the past and the future. 13. Their mindset is always changing and very creative According to research conducted by Schawbel (2014) 57 , namely the following: 1. Highly entrepreneurial spirit, savvy technological ability, relies heavily on social media, prefers online shopping, and is more pampered and influenced by parents of generations X and Y 2. They are independent, educated, and ready to face big challenges in the era of advanced technology 3. Free online education is preferred According to research conducted by Holroyd (2015) 58 discussing the alpha generation from an educational point of view, namely the following: 1. Most formally educated, attending education early, and learning longer because of online learning 2. Materialistic	Generations exposed to technology early on have critical thinking, are individualistic, and have high technological skills. They are also inclined to leadership and experience changes in lifestyle and consumption preferences
3	Kearifan Menyikapi Anak Usia Dasar di Era Generasi Alpha	Assingkily et al 13	Literature study	Examining the wisdom of addressing the alpha generation phenomenologically	According to a child development expert from the University of Indonesia, Anastasia Satryo: 1. Generation Alpha digital literacy 2. Not familiar with the process Other characteristic findings: 1. Have more critical thinking 2. Growing individualistic or antisocial	Generation Alpha grew up in an environment highly exposed to digital technology. They have easy access to information and more critical thinking but tend to be individualistic or less socially engaged in the real world
4	An analysis of the preschool teachers view on alpha generations	Apaydin and Kaya 15	Qualitative research with content analysis	Identify the characteristics of alpha generation students from the perspective of PAUD teachers	1. More curious, rule-free, hot-tempered, mobile, and self-centered behavior than Generation Z 2. Have high self-esteem, and are more emotional and aware 3. Tend to experience technology addiction and egocentrism and have a tendency to violence 4. Use of tablets, cell phones, the internet, television, or digital media; tends to be closed in communication and behave more individually than Generation Z 5. High level of perception, enjoys music, uses numbers effectively, and tends to be cautious and emotional 6. May have difficulty communicating with friends and is more inclined to show leadership than sharing	Generation Alpha is a generation exposed to technology early on with distinctive behavioral characteristics, challenges, and differences from previous generations, such as Generation Z
5	Study on the alpha generation and the reflections of its behavior in the organizational environment	dos Reis 1	Literature study (bibliographic research methods)	Analyze the behavior of the alpha generation that focuses on the family and school environment by projecting how these characteristics can affect the organizational environment	1. Digital natives 2. Focus on creativity, dynamism, leadership, and a strong technological connection	This generation is a digital native or digital generation. They grow up with intense exposure to digital technology from an early age. The alpha generation has high creativity, is dynamic in dealing with change, shows leadership, and has a strong connection with technology
6	Generation alfa: understanding the next cohort of university students	Ziatdinov et al 4	Qualitative research with theoretical analysis	Provide recommendations on how universities can be changed to ensure a better learning experience for Generation Alpha students, consistent with ensuring a better learning experience for Generation Alpha students	According to research conducted by Nagy and Kölcsey (2017) 59 : 1. Social media has an immediate influence 2. Quick access to information 3. Selfish 4. Know no boundaries According to research conducted by Taylor and Hattingh (2019) 60 : 1. Apply online reading skills 2. Ability to interpret information 3. Social connection online 4. Learning through technology According to research conducted by Apaydin and Kaya 15 : 1. Lack of technological literacy 2. High level of perception 3. Like visual, auditory, and kinesthetic tools Alpha generation students' learning styles will be highly dependent on and connected to technology	Generation Alpha has individual leanings and relies more on technology as the main source of entertainment. They also have a special interest in technological devices that can support their experiences in playing and interacting with the digital world
7	Desain Perpustakaan Generasi Z dan Alpha (antara Regulasi Pendanaan dan Teknologi.	Nurdiansyah 9	Literature study	Seeing and explaining the aspects of regulation, funding, and technology in creating a technology-based library is a major concern so that it can be seen in terms of what needs to be improved so that the library can run and continue to grow according to the needs of the Z and alpha generations	1. They get more entertainment from technology than social experiences in the real world, so they tend to be individualists 2. They tend not to follow the rules and are more dominant in leadership 3. Exposure to various “Internet toys” supporting devices that include technologies such as image or speech recognition 4. They are heavily influenced by technological devices such as smartphones and tablets, video games, driverless trains, autonomous cars, and smart speakers	1. Generation Alpha is exposed to digital technology from an early age, tends to be individualistic, does not follow rules, and is dominant in leadership 3. Heavily influenced by technology devices such as smartphones, tablets, video games, and other smart devices
8	Generasi Alpha saatnya anak usia dini melek digital refleksi proses pembelajaran di masa pandemi COVID-19	Swandhina and Maulana 10	Literature study	Responding to various challenges at this time, especially various forms of parental concern about the various impacts of using technology for early childhood, especially during the COVID-19 period	According to McCrindle (Yeni, 2017) 61 , namely: 1. Most familiar with the Internet 2. Lack of socialization, creativity, and individualism cannot be separated from gadgets 3. Want instant things and lack respect for the process According to Dr. Neil Aldrin, M.Psi, Psychologist (Yeni, 2017) 61 , namely: 1. Be more pragmatic and materialistic 2. Think practically, pay less attention to values, and generally more selfish According to Family Guide Indonesia, Yeni 2017 61 , namely: 1. Most familiar with digital technology 2. Most intelligent compared to previous generations According to Purnama 2018, 62 namely: 1. Bossy, dominant, and likes to arrange 2. Don't like to share 3. Don't want to follow the rules 4. Technology becomes part of their life 5. Ability to communicate	1. High-tech skills and familiarity with the Internet 2. Tend to be individualistic, less friendly, and less creative 3. Want instant gratification and lack value in the process 4. Materialistic, pragmatic, and selfish 5. Smart and most skilled in the use of digital technology 6. Dominant, like to manage, and do not like to share 7. Less interested in rules and has limitations in direct communication
9	Generasi Alpha Tantangan dan Kesiapan Guru Bimbingan Konseling dalam Menghadapinya	Anwar 11	Literature study (library research)	Describes the character of children who are born as the alpha generation and the readiness that must be prepared by guidance and counseling teachers in dealing with them	1. Instant generation 2. Love freedom 3. High self-confidence 4. Desire to be acknowledged 5. Ease of information 6. Proficient in using gadgets 7. Their understanding of technology is faster and better than the previous generation	Generation Alpha has characteristics like instant things, love of freedom, high self-confidence, desire to be recognized, easy access to information, proficiency in using gadgets, and a better understanding of technology than the previous generation
10	Demand of preschool education by alpha generation on edutainment leisure in the city	Rusman et al 12	Literature study	Discusses the basic capabilities of cities that make them worthy as edutainment destinations with specific references on demand from preschool education in introducing the alpha generation	1. Based on research by dos Reis, 1 the alpha generation is “digital natives” 2. According to Barkowitz (2016), the alpha generation has relied heavily on display and touchscreens since its birth in the technological era 3. Based on Carter (2016) 63 , • Children grow up exposed to various technological features such as high-tech gadgets, the Internet, and social networks • Tends to be more influenced by information in visual and video formats compared to written and verbal formats 4. Based on Schawbel (2014): • High entrepreneurial spirit • Highly technology savvy and heavily dependent on social media • Prefer online shopping • Highly influenced by their parents • Able to meet their own needs and desires, more educated, and ready to face the big challenges in the future 5. From an educational perspective, Ramadlani and Wibisono 14 state several educational approaches for Generation Alpha focus on visual literacy and character education	Generation Alpha, who are “digital natives,” are exposed to and immersed in the digital world from the very beginning of their lives. They rely heavily on technology, particularly display and touchscreens. They are more influenced by visual information and videos and are highly entrepreneurial. They also tend to be well educated, up for a challenge, and influenced by their parents. In education, an approach that focuses on visual literacy and character education can be applied to Generation Alpha

General article characteristics in Table 2 include a summary of the 37 selected articles. Ten articles used randomized controlled trials, 16 17 18 19 20 21 22 23 24 25 8 randomized controlled clinical trials, 26 27 28 29 30 31 32 33 5 randomized clinical trials, 34 35 36 37 38 2 randomized crossover clinical trials, 39 40 2 systematic review, 41 42 1 prospective randomized study, 43 1 clinical trial, 44 1 randomized cluster trial, 45 1 quasi-experiment, 46 1 *in vivo* study, 47 1 single blinded crossover study, 48 1 randomized controlled crossover clinical study, 49 1 comparative study, 50 and 1 randomized interventional clinical study. 51 The complete characteristics of the article are listed in Table 2 .

**Tabel 2:** Presentation of a review of 37 articles examining the linkage of special characteristics of alpha generation children with behavioral management in dentistry

Sl. no.	Title	Study	Study design and sample	Objective	Assessment method	Behavior management	Result and conclusion
1	Decreasing disruptive behaviour during routine dental visits: a video modelling intervention for young children	Hine et al 16	Randomized controlled trial 40 children aged 3–6 y	To evaluate the results of an interdisciplinary collaborative project and the initial benefits of a practical video modeling intervention to reduce disruptive behavior in children	1. Demographic questionnaire 2. Direct observation 3. Subjective behavior rating scale	Providing video modeling	Video modeling can improve children's calmness and orderliness in dental health care without disrupting their routine and order of care
2	Distraction With virtual reality goggles in paediatric dental treatment: a randomised controlled trial	Zaidman et al 39	A randomized crossover clinical trial 29 children aged 4–12 y	To test whether the use of virtual reality (VR) goggles in routine pediatric dental care can reduce pain perception during local anesthesia and rubber dam placement	1. Wong–Baker Faces Pain Rating Scale (WBFPRS) 2. Modified Behavioral Pain Scale (MBPS)	The use of VR glasses	VR glasses may decrease pain perception during rubber dam placement in children, but have limited benefit during local anesthesia administration
3	The efficacy of little lovely dentist, dental song, and tell-show-do techniques in alleviating dental anxiety in paediatric patients: a clinical trial	Abbasi et al 44	A clinical trial 160 children aged 4–10 y	To determine the efficacy of the three technique: little lovely dentist, dental songs, and tell-show-do (TSD) in reducing dental anxiety, by measuring heart rate	1. Heart rate recording 2. Facial Image Scale (FIS)	1. Little lovely dentist application 2. YouTube (dental video songs) 3. Tell-shows-do	1. “Little lovely dentist” “dental songs” application can alleviate dental anxiety 2. TSD techniques do not show to be beneficial in reducing anxiety levels
4	Effects of psychological behaviour management programme on dental fear and anxiety in children: a randomized controlled clinical trial	Song et al 26	A randomized controlled clinical trial 48 children with average age 5.6 y	To develop a program that applies psychological behavior control theory and demonstrate the program's effect on fear and anxiety toward dental treatment	1. Heart rate recording 2. Procedure Behavior Checklist (PBCL) 3. WBFPRS	Information and Communications Technology (ICT) are videos in the waiting room and treatment room	The program is effective in relieving fear and anxiety and learning cooperative behavior
5	Efficacy of audiovisual distraction using eyeglasses during dental care: a randomized clinical trial	CustÓdio et al 27	A randomized controlled clinical trial 44 children aged 6–7 y (average age: 7.7 y)	To examine the efficacy of audiovisual (AVD) using VR goggles compared to conventional behavior management techniques during procedures requiring local anesthesia	1. Venham scale 2. The Brazilian version of the Face, Legs, Activity, Cry, and Consolability (FLACC) 3. Behavioral Pain Assessment Scale 4. Oximetry (measuring the pulse) 5. Faces Pain Scale-Revised (FPS-R scale) 6. Accelerometer	1. Audiovisual eyeglasses (AVE) 2. Conventional behavior management	AVE can be used as a distraction technique
6	Application of virtual reality on non-drug behavioral management of short-term dental procedure in children	Ran et al 17	A randomized clinical trial 120 children aged between 4 and 8 y	Measuring the role of VR distraction on behavior management in short-term dental procedures in children	1. The Children's Fear Survey Schedule-Dental Subscale (CFSS-DS) 2. WBFRS, respectively. 3. The Frankl Behavior Rating Scale (FBRS) 4. Heart rate measures	VR	The use of VR reduces children's anxiety, pain, and length of dental procedures, and increases children's compliance
7	Experiential learning for children's dental anxiety: a cluster randomized trial	Zhu et al 45	A cluster randomized trial 988 children aged 7–8 y	Develop a school-based experiential learning (EL) intervention and evaluate whether EL is effective in reducing AD in primary school children	1. Modified Children's Fear Survey Schedule-Dental Subscale 2. Blood pressures (BP) 3. Pulse rates (PR)	EL TSD	School-based EL interventions prior to dental visits are feasible and effective in reducing children's dental anxiety during PFS
8	Assessing an active distracting technique during primary mandibular molar pulpotomy (randomized controlled trial).	Alsibai et al 18	Randomized controlled trial 105 children aged 6–10 y	To evaluate the effectiveness of two different distraction techniques (audio video distraction/video game distraction) in the management of anxious pediatric patients during dental treatment	1. Pain assessment scale 2. Behavior assessment scale	1. Active distraction 2. Passive distraction 3. Basic behavior guidance technique	1. Video games through joysticks on tablets and headphones can relieve anxiety and dental pain during pulpotomy in children 2. Cartoon movies through tablets and headphones do not reduce pain
9	Effect of virtual reality distraction on pain and anxiety during infiltration anesthesia in pediatric patients: a randomized clinical trial	Felemban et al 19	Randomized controlled trial 50 children aged 6–12 y	To evaluate the effect of VR distraction on anxiety and pain during buccal infiltration anesthesia in pediatric patients	1. Pulse rates 2. WBFPRS	The use of virtual glasses or VR	VR glasses can be used in reducing anxiety and pain
10	Evaluation of children's pain expression and behavior using audio visual distraction	Delgado et al 20	Randomized controlled trial 100 children aged 4–6 y	To evaluate the effectiveness of overhead film devices on pain expression and behavior in children, aged 4–6 y during dental treatment	1. WBFPRS 2. FBRS	Providing AVD	AVD is effective during treatment in the workspace
11	Effect of virtual reality distraction on pain and anxiety during dental treatment in 5 to 8 year old children	Shetty et al 21	Randomized controlled trial 120 children aged 5–8 y	To examine the effect of VR distraction techniques on pain and anxiety in children aged 5–8 y during brief invasive dental treatment. Changes in salivary cortisol levels during the procedure were also evaluated, with and without the use of VR distraction	1. Questionnaire: Screen for Child Anxiety Related Disorders (SCARED) questionnaire 2. Salivary Cortisol ELISA kit (K210S, XEMA Co., Ltd.) used	1. Conventional behavior management 2. VR distraction	VR successfully reduces pain and anxiety during brief invasive dental treatments
12	Effectiveness of self-designed dental storybook as behavior modification technique in 5 − 7 year-old children: a randomized controlled study	Deshpande et al 22	Randomized controlled trial 380 children aged 5–7 y	To determine whether self-designed dental storybooks are effective in modifying the behavior of 5- to 7-year-old children during examination and treatment planning visits, followed by dental restoration visits	1. Pulse rate measurements 2. FIS 3. Venham drawing test	1. Behavior modification with storybooks 2. Behavior modification without storybooks	1. Self-designed storybooks can serve as a relatively simple and effective tool when used before dental procedures 2. The book also helps facilitate patient awareness and motivation to start taking care of their dental health, ensuring a better, cavity-free future 3. Patients' and their parents' expressions of positive opinions about the use of self-designed storybooks in the dental environment
13	The effect of virtual reality distraction on pain perception of children aged 7-9 years during anesthesia procedure with the jet injector in dental treatment	Kaswindiarti et al 46	Quasi-experimental 30 children aged 7–9 y	To determine the effect of distraction methods using VR on pain perception in children aged 7–9 y during anesthesia procedures using jet injectors in dental treatment	1. WBFPRS	VR distraction	VR distraction reduces pain perception in children aged 7–9 y during anesthesia procedures with jet injectors in dental treatment
14	The effect of an audiovisual distraction method on 6-10-years old children's behavior during dental treatment: a clinical trial	Muhammed and Noori 53	A randomized clinical trial 40 children aged on the average of 6 and 10 y	To compare the effectiveness of a novel approach, VR video glasses when combined with conventional local anesthesia (LA) against conventional LA injection alone during dental treatment	1. Visual analog scale (child self-report) 2. Visual analog scale (parent report)–VAS 3. Pulse rate and oxygen saturation SpO _2_ (physiological) 4. FBRS and Houpt's scale	LA with AV VR glasses and without AV VR	AVD methods are effective in reducing the pain and discomfort that arise when administering local anesthesia in dental treatment
15	A new experience for child in pediatric dental clinic during pulp therapy procedures with the Google Card board Device	Sharma et al 48	Single blinded crossover design 43 children aged 5–8 y	To evaluate the child's experience during the pulp therapy procedure with the use of Google Cardboard	1. Pulse rate 2. FLACC scale 3. Direct observation of facial reactions 4. Modified child dental anxiety scale (MCDAS[f]) questionnaire	Google Card board Device	VR distraction method using Google Cardboard Device is effective in reducing dental anxiety in children undergoing various pulp therapy procedures
16	Comparison of the efficacy of Jilo animation approach versus conventional Tell-Show-Do (TSD) technique on cooperation and anxiety levels of children during dental practice: a randomized controlled clinical trials	Sahebalam et al 28	A randomized controlled clinical trials 50 children aged 4–6 y	To evaluate the effect of modeling on the behavior of a sample population of Iranian children using animated films, which simulate a real dental clinic environment with animated characters To compare the efficacy of this method with conventional TSD techniques	1. Venham Clinical Anxiety Scale (VCAS) 2. Venham Clinical Cooperation Scale (VCCS)	1. Jilo animation approach 2. Conventional TSD technique	Animated film modeling techniques can produce the desired effective effect during previsit preparation and dental treatment sessions involving children aged 4–6 years. This technique can be used with the conventional TSD technique to produce a positive synergistic effect
17	Effectiveness of intellectual color game, audiovisual and stress ball distraction methods on gagging and anxiety management in children	Linthoingambi et al 38	Randomization clinical trial 108 children aged 5–12 y	To evaluate the results of intellectual color play, AVD and stress ball distraction methods for reducing choking and anxiety in children	1. Postoperative gagging score 2. Postoperative anxiety score	1. Intellectual color game 2. AV 3. Stress ball	1. Intellectual color, AV, and stress ball games can be used as a distraction method to reduce vomiting and anxiety in children 2. The stress ball distraction was the most effective among the three methods used in this study 3. The stress ball distraction method can be recommended as an economical distraction method
18	Comparison of audio and audiovisual distraction techniques in managing the pain and dental anxiety during infiltration anesthesia injection in children: randomized clinical trial	Danesvar and Mazloumi 37	Randomized clinical trial 30 children aged 4–10 y	To compare the effect of audio and AVD techniques on anxiety and dental pain when injecting infiltration anesthetics in children	1. FLACC behavioral anxiety/pain assessment scale 2. FIS	Audio, AV and conventional behavior management	3D AV glasses can be an effective tool for reducing anxiety and perception of pain during injections
19	The use of a dental storybook as a dental anxiety reduction medium among pediatric patients: a randomized controlled clinical trial	Alsaadoon et al 29	A randomized controlled clinical trial 88 children aged 6–8 y	To evaluate the effectiveness of a specially designed dental storybook in reducing dental anxiety and improving children's behavior during examination visits and treatment plans, followed by restorative dental visits	1. Children's Fear Survey Schedule-Dental Subscale (CFSS-DS) 2. VCAS 3. FBRS	Use of dental storybooks	Preparing the child with a dental storybook before the visit reduces anxiety and improves behavior during dental treatment
20	Comparative evaluation of effectiveness of tell-play-do, film modeling and use of smartphone dental application in the management of child behavior	Kevadia et al 30	Randomized controlled clinical trial 75 children aged 6–9 y	To evaluate the effectiveness of three behavior modification techniques; TPD, film modeling, and smartphone dental apps in managing child behavior during dental practice	1. Pulse rate score (Heart Rate) 2. FIS 3. Venham pictorial index (VPI)	1. Tell-play-do 2. Film modeling 3 Dental application on smartphone	1. The tell-play-do technique effectively and efficiently reduces children's fear and anxiety about dental treatment. Tell-play-do can be a functional alternative to TSD techniques and modeling during dental treatment 2. The tell-play-do technique is more efficient for controlling the anxiety of children aged 6–9 years to achieve more cooperative behavior during dental treatment 3. Smartphone dental applications can be used as an adjunct to conventional behavior modification techniques
21	A comparison of audio and audiovisual distraction techniques in managing dental anxiety in pediatric patients: a clinical study	Mishra et al 49	Randomized controlled crossover clinical study 100 children aged 4–10 y	To compare audio and AVD techniques in managing anxiety and dental pain in children	1. The Venham anxiety scale 2. Pulse measurement 3. Measurement of oxygen saturation 4. WBFPRS	1. Audio 2. AV	AV and audio distraction techniques can be used effectively to treat children
22	Effect of virtual reality glasses distraction on the anxiety of preschool children during pulpotomy treatment (randomized controlled clinical trial)	Mahmoud et al 31	Randomized controlled clinical trial 44 children aged 4–5 y	To evaluate and compare the effect of distraction VR glasses with conventional behavior management techniques on children's dental anxiety during pulpotomy treatment	1. Venham clinical anxiety rating scale 2. Cortisol changes in saliva	1. VR glasses 2. Conventional behavior management techniques	VR glasses are useful in managing dental anxiety in preschool children, especially during intraoral examinations
23	Effectiveness of aromatherapy and music distraction in managing pediatric dental anxiety: a comparative study	James et al 50	A comparative study 150 children aged 6–8 y	To compare and evaluate the efficacy of aromatherapy using citrus essential oil with music distraction in the management of anxious pediatric dental patients	1. Venham's picture test (VPT) 2. FIS	1. Aromatherapy 2. Music distraction	Music and aromatherapy, or a combination of the two, can be used as a behavior management technique in the dental clinic to reduce pediatric patient anxiety and make dental visits an enjoyable experience for patients, patient's parents' and dentists
24	Effectiveness of smartphone application in reducing anxiety during dental procedures: a randomized controlled trial	Derbala et al 23	Randomized controlled trial 38 children aged 6–8 y	To evaluate the effectiveness of a smartphone application (TPD) in reducing preoperative anxiety in children undergoing restorative treatment with dental local anesthesia	1. VPT 2. Heart rate (HR)	1. Smartphone application 2. Traditional behavior management techniques (TSD)	1. TSD and TPD techniques can reduce dental anxiety in children aged 6–8 y 2. Greater anxiety reduction was achieved using the “Smartphone App” intervention as the TPD technique compared to the TSD technique
25	Comparison of virtual reality glasses vs on-screen distraction technique in reduction of pediatric dental anxiety: an in vivo study	Tailor et al 47	An in vivo study 40 children aged 4–8 y	To assess the effectiveness of VR headsets and tablet-on-screen techniques in anxious pediatric patients during dental treatment	1. Physiological parameters; HR, pulse rate, and oxygen saturation 2. Measurement of behavior; Venham's clinical anxiety rating scale, VPT	1. Distraction VR glasses 2. Onscreen distraction	AV aids are an effective alternative to tablets (onscreen diversions) in managing anxious children in the dental office
26	Comparison of three behavior modification techniques for management of anxious children aged 4-8 years	Radhakrishna et al 51	Randomized, interventional, clinical study 60 children aged 4–8 y	To compare the effectiveness of Tell-Show-Play-doh, a smartphone dentist game and conventional TSD techniques in reducing dental anxiety among children aged 4–8 y	1. Pulse rate 2. FIS 3. FBRS 4. FLACC behavior scales 5. Validated questionnaire	Group 1: Tell-Show-Play-Do Group 2: smartphone dentist game Group 3: TSD techniques	The Tell-Show-Play-doh technique and smartphone dentist game effectively reduce dental anxiety in pediatric patients
27	The effect of different non-pharmacological methods in the management of pediatric patients' dental anxiety and behaviour, a randomized control study	Ghibban et al 24	A randomized control study 42 children aged 5–12 y	To assess the effect of different nonpharmacological methods on the management of anxiety and behavior of pediatric patients during dental treatment	1. FIS 2. Child finger pulse oximeter	1. TSD, 2. AVD 3. Presence of parents in the treatment room	No significant difference was observed between the three nonpharmacological techniques. However, according to the FIS score and pulse rate results, TSD is the most accepted method for children. The main reason for dental fear and anxiety is dental injection
28	Effectiveness of cognitive behavioral play therapy and audiovisual distraction for management of preoperative anxiety in children	Rajeswari et al 36	Randomized clinical study 45 children aged 6-10 y	To evaluate the effectiveness of cognitive behavioral play therapy and AVD for managing preoperative anxiety in children	1. Pulse oximeter (HR measures) 2. FIS	1. Cognitive behavioural play therapy (CBT) 2. AVD 3. TSD (control group)	Active distraction with cognitive behavioral play therapy reduced preoperative anxiety in children more effectively than AVD and TSD techniques
29	Audio visual distraction effect on heart rate in children during dental treatment, a randomized clinical trial	Zakhary et al 32	A randomized controlled clinical trial 42 children aged 5–8 y	To determine the effect of AVD on children's heart rate during dental treatment	1. Pulse oximeter (HR measures)	1. Conventional TSD 2. AVD	AVD can effectively reduce anxiety during dental treatment and help patients enjoy dental visits
30	Effectiveness of audio visual distraction using virtual reality eyeglasses versus tablet device in child behavioral management during inferior alveolar nerve block	Al-Halabi et al 35	Randomized clinical trial 102 children aged 6–10 y	To evaluate the effectiveness of two different AVD techniques (“VR box” AV glasses vs. tablets) in the management of anxious pediatric patients during inferior alveolar nerve block (IANB)	1. FLACC scale	1. AVD using AV glasses 2. Use of tablet devices	1. Video viewing on tablet devices provides the best results in relieving anxiety and dental pain during IANB in children 2. Although the use of “VR box” AV goggles does not have an additional advantage in most children, it is more acceptable in patients aged 8–10 y than younger ones and provides children with some interesting experiences that can lead to better behavior at future dental visits
31	Behaviour management of the contemporary child in paediatric dentistry: an overview of the research	da Silva et al 41	Systematic literature study 17 articles	To provide an overview of the most relevant studies on nonpharmacological behavior management techniques for contemporary children, the so-called alpha generation, undergoing dental treatment	Systematic literature study	Systematic literature review	Children today are born and live surrounded by technology and have a new view of the world, meaning that the use of interactive screens and AV glasses is paramount. Therefore, it is imperative to use these devices to distract and relax pediatric patients during dental treatment
32	Effect of a relaxation training exercise on behaviour, anxiety, and pain during buccal infiltration anaesthesia in children: randomized clinical trial	Sridhar, et al 33	A randomized controlled clinical trial with a parallel group 66 children aged 7–11 y	To evaluate the effects of this relaxation exercise (bubble breath) on dental anxiety, dental behavior, and pain intensity during local anesthetic buccal infiltration in children	1. FBRS 2. FIS 3. Pulse rate 4. WBFPRS 5. FLACC scale	The use of bubble breath	The use of bubble breath exercises is beneficial in reducing pain felt during maxillary buccal infiltration under anesthesia
33	The effect of television distraction versus Tell-Show-Do as behavioral management techniques in children undergoing dental treatments	Kharouba et al 43	A prospective randomized study 69 children with the average age of 6.8 y (5–12 y)	To evaluate the effects of watching television during dental treatment on pediatric patients' anxiety and cooperation compared to commonly used and conventional TSD behavior management methods	1. FIS 2. FBRS	Television and TSD	Television distraction is an effective and inexpensive method to educate anxiety and increase cooperative behavior in children during dental treatment
34	Effectiveness of two different behavioral modification techniques among 5–7-year-old children: a randomized controlled trial.	Vishwakarma et al 25	A randomized controlled trial 98 children aged 5–7 y	To evaluate the effectiveness of direct modeling compared to the TPD technique among children aged 5–7 years, with the null hypothesis stating there is no difference between the two behavior modification techniques	1. Heartbeat a. FIS 2. Index a. Venham 6-point	Tell play do Live modeling procedure	TPD is effective in reducing children's fear and anxiety about dental treatment
35	The effect of breathing exercise using bubble blower on anxiety and pain during inferior alveolar nerve block in children aged 7 to 10 years: a crossover randomized clinical trial	Bahrololoomi et al 40	A crossover randomized clinical trial 35 children aged 7–10 y	To evaluate the effect of breathing exercises using a bubble blower on anxiety and pain during IANB in children aged 7–10 y	1. FIS, blood pressure, and pulse 2. FLACC scale 3. BFPRS	1. Breath Exercise (bubble blower) 2. Without breath exercise in IANB	Breathing exercises using a bubble blower can be an efficient distraction and relaxation method to reduce pain in children aged 7–10 y with moderate to severe anxiety during IANB
36	Behaviour and anxiety management of paediatric dental patients through virtual reality: a randomised clinical trial	Gómez-Polo et al 34	A randomized clinical trial 80 children aged 5–10 y	To assess the effectiveness of using VR headset as a distraction technique to reduce anxiety and improve children's behavior during dental treatment	1. FIS 2. Test Frankl	AV VR TSD	Use of a VR headset during dental treatment significantly reduces anxiety
37	Systematic review and meta-analysis of virtual reality in pediatrics: effects on pain and anxiety	Eijlers, et al 42	Systematic literature review	To gather evidence on the effectiveness of VR as a distraction or exposure tool, compared to standard care, on pain and anxiety in pediatric patients undergoing medical procedures	–	Systematic literature review	Pediatric patients undergoing various medical procedures benefit from VR as a tool to reduce pain and anxiety

## Discussion

In dental practice, understanding the unique characteristics of the alpha generation child is essential in providing adequate care and a positive experience, including considering the psychological aspects. These aspects are essential factors that must be considered when treating alpha generation children in dentistry. Alpha generation children also have high self-confidence but may experience anxiety or discomfort in dental care situations. 16 Our research reveals that symptoms such as anxiety, fear, and stress can appear in children, which have an impact on quality of life, complicate dental treatment, and even cause children to become uncooperative, which may be caused by “painful dental surgery” such as seeing instruments in the form of long needles for injections. 8 41 Therefore, a team of dentists or practitioners in dentistry needs to have in-depth knowledge about the characteristics of the alpha generation in creating a supportive, child-friendly environment and empathy, which are helpful for effective communication, reduce the anxiety that may arise, and increase the comfort of the alpha child during treatment.

### Special Characteristics of Alpha Generation Children in General

This research will show an overview of the special characteristics of the alpha generation children, which are presented in Table 1 . Overall, the findings of this literature study indicate that the characteristics of the alpha generation children are related to technological advances. Fadlurrohim et al 8 observed that the alpha generation is closely connected to digital technology and has intelligent characters. This is supported by the fact that children of the alpha generation are the generation exposed to digital technology from an early age, so they are called digital natives or digital generation, which makes them have critical thinking, individualism, high technological skills, are dynamic toward change, and tend to show leadership. 9 10 12 13 15 37 They depend on technology, especially gadgets and touchscreens. 16 37 Closeness to this technology gives them easy access to information, which can affect their ability to analyze and evaluate the information they receive so that they have more critical thinking. 15 However, they have challenges and differences with previous generations, such as dependence on gadgets and deficiencies in social interaction, for example, reduced interaction outside the home. 9 14 Affected by the role of technology in their lives, technology becomes an integral part of how they interact, learn, and express. The existence of developments and advances in technology create the characteristics of the alpha generation of children.

Negative characteristics in several studies, such as tending to be individualistic or less socially interacting in the real world, are an actual result of technology dependence, which is supported by the fact that they rely more on technology as the primary source of entertainment. They are also particularly interested in devices that support their experiences in playing and interacting with the digital world. 12 15 Another study states that this generation is less sociable and creative and wants instant gratification by not appreciating the process that makes them known as the instant generation. 12 16 They are said to be dominant, controlling, and dislike sharing. This generation is less interested in rules and has limited direct communication. 12 The research by Berkowitz (2016) 13 cited in the literature study by Ramadlani and Wibisono 14 observed that children of this generation are active, tend to break rules, run away from all crowds, and like to disturb crowds of events. The negative characteristics in these articles can be a challenge and an obstacle in implementing child behavior management in dentistry.

Other positive characteristics found in several studies besides their proficiency in using technology are that they have high self-confidence, are independent, and have a high entrepreneurial spirit. 16 They are well educated, ready to face challenges, and are still influenced by their parents. Their preferences and ways of learning are also closely related to technology. The information they get is influenced by visual and audio information. This is supported by research that education that focuses on visual literacy and character education can be applied to the alpha generation. In pediatric dentistry, these positive characteristics support dentists' efforts to optimize the management of children's behavior, especially in the alpha generation. 37

As shown in Table 1 , the salient characteristics of this generation's children are their ability, understanding, and acceptance of high technology and independence because they can adapt to technological changes, like instant things, and have less respect for processes compared to previous generations. 29 34 52 These characteristics have essential implications in dentistry, including their influence on communication and adaptation of the behavior management of alpha generation pediatric patients, especially in their care in dentistry.

### Alpha Generation Behavior Management in Dentistry

As shown in Table 2 , the authors collected studies in the field of dentistry that have patient descriptions of the criteria for alpha generation children who have the potential to be cooperative. The studies addressed anxiety and perception of pain associated with disruptive behavior during visits, including excessive movement, verbal distractions, and disobedience. Therefore, a psychological approach must be applied to build communication and trust between patients and practitioners in reducing stress, fear, and anxiety. 8 The techniques found in the literature have shown promising results because they are scientifically accepted and effective. The techniques reported in the previous literature are tell-show-do (TSD) techniques, positive reinforcement techniques, modeling techniques, and other techniques. 8 27 Therefore, knowing behavior management techniques suitable for alpha generation children is essential.

This research is interested in changes in modification of behavior management techniques that may appear in the field of dentistry, considering that the focus of this research is the alpha generation of children who have been close to technology since birth. A study in 2013 showed that behavior management techniques related to using technological distraction methods, namely, audiovisual glasses, are widely studied. According to CustÓdio et al, 27 using audiovisual glasses is a pleasant distraction method without side effects. This may be because their focus is diverted from watching cartoons, and the child's anxiety is reduced because they are already familiar with the environment and the procedures to be carried out. This is in line with the findings of a systematic literature study and meta-analysis by Eijlers et al, 42 which aimed to find evidence of the effectiveness of VR as a distraction or as a means of distraction compared to other standard methods. They observed that audiovisual virtual reality (VR) glasses can reduce the perception of pain and anxiety in pediatric patients undergoing medical procedures. 17 20 42 52 These changes made treatment time shorter. However, it was also reported that the perception of pain decreased only when the placement of the rubber dam only had limited benefits during the administration of local anesthesia. 17 A study conducted by Delgado et al found that the distraction of audiovisual glasses was unrelated to the perception of pain in children. However, this technique significantly increases cooperation in treatment because it increases the child's attention to the device used. 20 This differs from the study of Felemban et al, 19 which showed that VR glasses have the additional benefit of reducing pain and anxiety when injecting local anesthetics.

Several other studies have also found that audiovisual glasses can be used because they reduce the perception of pain and anxiety during various treatments such as intraoral examinations, invasive treatments, and local anesthetic injections. 24 29 31 32 35 36 46 47 48 49 53 It has been reported that the audiovisual spectacle technique results in high satisfaction, pain reduction, and a degree of cooperation, which lead to a positive attitude during treatment. The study of audiovisual glasses also stated that VR audiovisual glasses function effectively as a distraction clinically, are practical, safe, and do not require prior training for dentists or practitioners. Audiovisual spectacle distraction was found to be effective compared to standard methods used in dentistry. 21 Most of the studies also revealed decreased anxiety at subsequent visits after applying audiovisual behavior management techniques at the first visit. 24 29 31 32 35 36 46 47 48 49 53 This is because they gained acceptance mechanisms and learned to discriminate between procedures that generate tension and those that do not. In a study conducted by Linthoingambi et al, 38 it was revealed that audiovisual glasses were also effective but not as effective as stress balls in reducing the effects of nausea and anxiety. This limitation of the audiovisual technique was reported by Mahmoud et al, 31 who observed that no VR glasses adapted for children available. Another limitation was found in the study by Al-Halabi et al 35 in Syria, who reported that using the VR Box audiovisual distraction technique was more difficult because it hindered the practitioner from seeing intraorally and obstructed the child's vision, thus increasing children's anxiety about the surrounding environment. A systematic literature study revealed that using interactive screens and audiovisual technology is essential for distracting the patient's attention and relaxing the patient during treatment. Mishra et al 49 reported that the effectiveness of audiovisual distractions did not differ significantly from that of audio distractions during treatment. Audiovisual distractions are effective as a modified behavior management method for treating alpha generation children.

Then, there are modeling techniques using technology, namely, videos by several authors. These videos were created by dentists using existing technology of audio visual. The videos had dentists and dental assistants as models walking into the treatment room and undergoing a short exposure to each of the main components of dental examination and cleaning. Video modeling was given to the same treatment group while in the waiting room, as reported in a study in Indonesia. 54 The results showed that video modeling could relieve fear and anxiety, increasing calm and orderliness among children during treatment. Video modeling with cartoons also produced the desired practical effect during a series from the beginning to the end of treatment. Similar to television distraction, a research by Kharouba et al 43 reported that this method was more effective for reducing anxiety and increasing cooperative behavior than TSD. Television as a distraction is said to be a method that requires minimal maintenance and is low-cost compared to other distraction methods. 49 In line with the research by Abbasi et al, 44 they reported that playing dental video songs via YouTube can relieve children's dental anxiety. A study by Kevadia et al 30 also stated that film modeling effectively reduces children's fear and anxiety. However, the tell-play-do (TPD) technique is observed to be more efficient in controlling the anxiety of children aged 6 to 9 years. This is in line with the findings of Vishwakarma et al 25 who reported that TPD was more practical than direct modeling on anxiety levels and that it increased cooperative behavior during treatment of the mandibular teeth in children aged 5 to 7 years. 23 Video modeling is effectively used in behavior management of alpha generation children, both manually and from existing applications. 54

Several studies conducted in Pakistan, Egypt, and India have also reported using smartphone applications to reduce dental anxiety levels. 22 30 51 Smartphones can be used to open game applications that are used to reduce anxiety. 22 51 This cross-sectional studies found that the presence or absence of parents did not affect the increase in positive behavior during visits. 32 From this study, the authors emphasized that the results are consistent with many studies on effective ways to reduce stress and fear in children in the face of types of treatment and dentists. Several other behavior management techniques were found, such as making bubble breathing exercises, bubble blower, bubble breath, and provision of dental storybooks, carried out before treatment to effectively and efficiently reduce anxiety, improve behavior, and reduce pain perception. 34 40 It should be noted that other techniques, such as using aromatherapy, distraction with music, and giving dental storybooks with conventional TSD techniques, can also increase positive behavior in pediatric patients. However, they are less effective than other modern techniques such as audiovisual, use of smartphone applications, video modeling, and others found in this study's entire population.

Before using these behavior management techniques, practitioners continue to communicate effectively, such as providing procedural and sensory information about what is honestly expected to create a sense of trust in the alpha generation of children. 41 The 2021 American Academy of Pediatric Dentistry (AAPD) guidelines on behavior for the pediatric dental patient provide behavioral guidance to build communication and mutual trust between dentists/staff and children/parents in providing quality dental and oral health care in a comfortable, safe, and effective manner. 55 The basic behavioral guidance expressed in the 2021 AAPD guidelines is through communication, such as active/reflective listening, which can help build rapport and trust. The teacher–student technique can be applied, and once the procedure is started, two-way communication must be maintained. The dentist makes the child a good, active participant and becomes caring. With this two-way communication, dentists can provide one-way behavioral guidance through directions. Guidance or communicative guidance is said to consist of several special techniques that, when integrated, will enhance the evolution of cooperative patients. This communicative technique is an ongoing technique that becomes an extension of the dentist's personality.

Regarding this communicative technique, the dentist can consider the patient's progress in determining and applying the behavior management technique. 27 A relationship of mutual trust and appropriate behavior management will create a comfortable environment in the future, especially if there is continued treatment. This is supported by a research by Song et al 26 who observed that video technology makes pediatric patients more cooperative and comfortable after their next visit. Several studies reveal that appropriate behavior management techniques provide long-term effects until the next visit regarding cooperativeness. Before carrying out appropriate behavior management techniques, it is necessary to have communication and efforts to build trust in children. 23 25 43

### Linkage of Findings on Special Characteristics of Alpha Generation Children and Behavioral Management in Dentistry

This study aims to look for the unique characteristics of the alpha generation children in general and their relation to dentistry, especially in their behavior management. The findings are presented in Tables 1 and 2 . The authors found a correlation or link between the findings in Tables 2 and Table 1 . The most prominent behavior management found in Table 2 was the use of technology. Audiovisual distraction techniques, audio modeling, game applications, and smartphone use are used in behavior management and have been proven effective in most studies. This is related to the results of the analysis of the findings from Table 1 that the unique characteristics of the alpha generation that stand out from the children of this generation are their ability, understanding, and acceptance of high technology and independence because they can adapt to technological changes compared to previous generations.

The characteristics of alpha generation children give them a better understanding of blending in with existing changes. Alpha generation children are very familiar with technology, such as using smartphone game applications and consuming information or audiovisual content through digital media, which creates a desire for everything instant and less respect for the process. As shown in Table 2 , technology and audiovisual tools such as VR glasses and video modeling can support the behavior management of alpha generation children. For example, video modeling can eliminate fear and anxiety and increase calm and regularity in child care. 28 41 Besides smartphones, the game applications listed in Table 2 can also be used as supporting tools in behavior management. Game application findings in research, such as health games and specially designed applications such as the Little Lovely Dentist application, function to provide interactive experiences and educate them in maintaining their dental health. 44 These game applications are designed using existing techniques and theories to make them interested so they receive the procedure correctly and efficiently. Given the link between technologies such as audiovisual, video modeling, smartphones, and game applications and the unique characteristics of the alpha generation, dentists can utilize and integrate each element in their behavior management strategy to increase positive attitude and acceptance of the alpha generation in their lives—clinics to provide a supportive, child-friendly, effective, and enjoyable care environment.

The limitation of this study is that there is no risk-of-bias assessment, as for other things that are suggested for further research, namely, being able to compare the characteristics of children of the alpha generation with the previous generation in terms of characteristics and behavior related to behavior management in dentistry.

## Conclusion

The unique characteristics of the alpha generation children that stand out the most in this study are their understanding and acceptance of technology resulting from increased exposure to technology and consumption habits of digital media, as well as their character of not appreciating the process and wanting instant things.

An in-depth understanding of these characteristics is one of the keys to designing effective communication strategies and adjusting appropriate behavior management to create a trusting relationship, a pleasant environment, acceptance of the alpha generation, and maintaining the quality of dental care for the alpha generation. This is important for communicating and adapting their behavior management in the dental environment. Audiovisual technology, video modeling, smartphones, game applications, and appropriate strategies such as open and interactive communication in dentistry can be applied to support their behavior management.
